# Clinical Factors Associated with Documented Radiographic Pneumonia in Adults with Human Metapneumovirus Infection: A Hospital-Wide Cohort Study in Japan

**DOI:** 10.3390/jcm15208025

**Published:** 2026-10-16

**Authors:** Takeshi Saraya, Junki Tashiro, Hajime Furuno, Yoshitaka Sakamoto, Kei Nakajima, Sho Yamada, Takatora Akizawa, Narishige Ishikawa, Hiromi Ishida, Tatsuya Shirai, Jumpei Aso, Nozomi Kurokawa, Fumi Kobayashi, Yasuo Nakamoto, Manabu Ishida, Mitsuru Sada, Keitaro Nakamoto, Saori Takata, Haruyuki Ishii

**Affiliations:** Department of Respiratory Medicine, Kyorin University School of Medicine, 6-20-2 Shinkawa, Mitaka 181-8611, Tokyo, Japan

**Keywords:** human metapneumovirus, adult, pneumonia, C-reactive protein, auscultation

## Abstract

**Background/Objectives**: Human metapneumovirus (hMPV) is increasingly recognized as an important cause of adult respiratory infection, but data on pneumonia remain limited. We evaluated clinical characteristics, temporal detection patterns, and clinical factors associated with radiographic pneumonia among adults with laboratory-confirmed hMPV infection. **Methods**: We conducted a single-center retrospective observational study at Kyorin University Hospital, Tokyo, Japan, from December 2020 to February 2026. Adults aged ≥18 years with hMPV detected by multiplex polymerase chain reaction were included. The primary outcome, documented radiographic pneumonia, was defined by newly identified pneumonia-compatible parenchymal abnormalities on chest radiography and/or chest computed tomography (CT). Logistic regression was used to identify clinical factors associated with this outcome. **Results**: Among 590 hMPV-positive patients, 192 adults were included; 121 were managed as outpatients and 71 were hospitalized. Pneumonia was identified in 68 adults, including 43 of 71 hospitalized patients. Adult hMPV detection shifted from summer predominance in 2022–2023 toward increased spring and winter detections in 2024–2025 after the onset of the coronavirus disease 2019 (COVID-19) pandemic. By 2025, pneumonia was identified in 24.0% of outpatients and 65.5% of hospitalized patients. In the primary multivariable model, higher C-reactive protein (CRP) concentrations (adjusted odds ratio per 1 mg/dL, 1.12; 95% confidence interval (CI), 1.03–1.22; *p* = 0.008) and abnormal auscultation (adjusted odds ratio, 3.45; 95% CI, 1.21–9.83; *p* = 0.021) were independently associated with pneumonia. **Conclusions**: Adult hMPV infection was frequently associated with radiographic pneumonia, particularly among hospitalized patients. Elevated C-reactive protein concentrations and abnormal auscultation may help identify adults with hMPV infection who are more likely to have pneumonia.

## 1. Introduction

Human metapneumovirus (hMPV), first identified in 2001, causes upper and lower respiratory tract infections across all age groups [1]. Although traditionally studied in children, hMPV also causes clinically important disease in adults, especially older adults and those with immunocompromised states or cardiac or pulmonary diseases [2,3,4]. hMPV is an important cause of adult community-acquired pneumonia, with a clinical burden similar to that of respiratory syncytial virus infection [5,6]. Despite this burden, adult hMPV-associated pneumonia remains insufficiently characterized. In Japan, previous studies have described long-term care facility outbreaks and adult community-acquired pneumonia [7,8,9], but most focused on outbreaks, hospitalized patients, or established pneumonia. Thus, clinical factors associated with documented radiographic pneumonia among adults with laboratory-confirmed hMPV infection in routine care remain unclear. We therefore conducted a single-center, retrospective observational study to characterize adult hMPV infection, temporal detection patterns, documented radiographic pneumonia frequency, and factors associated with documented radiographic pneumonia and severe disease. 

## 2. Materials and Methods

### 2.1. Study Population

This single-center, retrospective observational study was conducted at Kyorin University Hospital, Tokyo, Japan. All patients who tested positive for hMPV between December 2020 and February 2026 via multiplex polymerase chain reaction using the FilmArray respiratory panel (BioFire Diagnostics, LLC, Salt Lake City, UT, USA) were screened. Patients aged <18 years were excluded from the adult cohort, and their data were summarized descriptively to contextualize the overall institutional burden of hMPV infection. Primary analyses were restricted to adults aged ≥18 years.

This study was designed to evaluate the clinical characteristics, temporal detection patterns, documented radiographic pneumonia burden, and clinical factors associated with documented radiographic pneumonia among adults with hMPV infection.

### 2.2. Data Collection

The clinical data were retrospectively extracted from the electronic medical records. Collected variables included age, sex, care setting, admitting department, activities of daily living, comorbidities, clinical symptoms, vital signs, laboratory findings, microbiological results, auscultatory findings, documented radiographic pneumonia status, hospitalization, length of stay, and disease severity.

The comorbidities included chronic lung disease, chronic obstructive pulmonary disease, asthma, cardiovascular disease, diabetes mellitus, chronic kidney disease, and malignancy. The clinical symptoms included fever, cough, sputum production, dyspnea, and hypoxemia. Laboratory variables included white blood cell count, neutrophil and lymphocyte percentages, C-reactive protein (CRP), lactate dehydrogenase, and albumin concentrations, and other biochemical parameters.

### 2.3. Definitions

Documented radiographic pneumonia was defined as newly identified pneumonia-compatible pulmonary parenchymal abnormalities on chest radiography and/or chest computed tomography (CT) during the hMPV episode. Chest radiography and CT findings were assessed separately. For each modality, the examination was considered not to have been performed when no imaging result was available in the chart abstraction. When the examination had been performed but no abnormal findings were documented, it was classified as imaging performed without abnormal findings. Pneumonia-compatible findings included infiltrates, nodular opacities, ground-glass opacities, consolidation, tree-in-bud nodules, or combinations of these findings. Isolated pleural effusion, isolated bronchial wall thickening, and isolated linear opacities were not classified as pneumonia-compatible in the primary definition. Patients with no available chest radiography or CT result and no clinical diagnosis of pneumonia documented in the medical record were classified as having no documented radiographic pneumonia in the primary analysis. A sensitivity analysis restricted to patients who underwent chest radiography and/or chest CT was performed to address potential verification bias; a separate sensitivity analysis used any abnormal chest imaging finding as an alternative pneumonia definition.

Severe pneumonia was defined as an A-DROP (age, dehydration, respiratory failure, orientation disturbance, and low blood pressure) score of ≥3. A-DROP was assessed using clinical and laboratory data recorded at the initial evaluation for the hMPV episode. One point was assigned for each of the following: age ≥70 years in men or ≥75 years in women; dehydration, defined as blood urea nitrogen ≥21 mg/dL; respiratory failure, defined as peripheral oxygen saturation (SpO2) ≤ 90% or arterial oxygen partial pressure (PaO2) ≤ 60 mmHg; orientation disturbance; and systolic blood pressure ≤90 mmHg. A score of 3 indicates severe disease in the Japanese A-DROP classification [10].

CRP concentration was analyzed as a continuous variable in the primary multivariable logistic regression model. Because a threshold near 2 mg/dL was derived from the present dataset, CRP ≥ 2 mg/dL was evaluated only in secondary and exploratory analyses.

Abnormal auscultation was defined as documented adventitious lung sounds on physical examination. Records without an auscultation assessment were treated as missing and were excluded from analyses requiring auscultation data. When auscultation was documented and no adventitious lung sounds were recorded, the examination was classified as no abnormal auscultation. For subtype analyses, any finding containing crackles was assigned to the crackle-predominant category; this category therefore included coarse crackles, fine crackles, coarse crackles/wheezes, and coarse crackles/rhonchi. Wheeze/rhonchi-predominant sounds included wheezes or rhonchi without crackles.

Pathogen profiles were classified into three mutually exclusive categories: hMPV alone, hMPV with other viruses, and hMPV with bacteria. “hMPV with other viruses” indicated viral codetection without bacterial coinfection, whereas “hMPV with bacteria” indicated bacterial coinfection irrespective of viral codetection. Bacterial coinfection was defined as clinically relevant bacterial detection from blood cultures or respiratory specimens, including good-quality sputum specimens (Geckler grades 4 or 5) judged clinically relevant by treating physicians.

The seasons were spring, summer, fall, and winter. Calendar years and seasons were used to evaluate the temporal and seasonal distributions of adult hMPV-positive cases. Data from 2026 were excluded from the temporal trend analyses because only data for January and February were available.

### 2.4. Outcomes

The primary outcome was documented hMPV-associated radiographic pneumonia based on pneumonia-compatible imaging findings. The secondary outcomes included hospitalization, length of stay, temporal trends in adult hMPV-positive detections, seasonal distribution, documented radiographic pneumonia proportion by care setting, and severe pneumonia (defined by an A-DROP score of ≥3).

We also evaluated the associations of CRP elevation and abnormal auscultation with documented radiographic pneumonia using an exploratory 4-group classification: neither CRP concentration ≥2 mg/dL nor abnormal auscultation, CRP elevation only, abnormal auscultation only, and both CRP elevation and abnormal auscultation.

### 2.5. Statistical Analysis

Continuous variables are presented as medians with interquartile ranges and were compared using the Mann–Whitney U test. Categorical variables are presented as numbers and percentages and were compared using the chi-square or Fisher’s exact test. Season-specific temporal trends from 2022 to 2025 were evaluated using Poisson regression models of observed case counts, with calendar year modeled as a continuous variable. Because no offset for the number of FilmArray tests or respiratory encounters was included, estimates are reported as count ratios rather than incidence rate ratios. Harmonic Poisson regression with annual sine and cosine terms was used to explore seasonality. Because only 16 season-year observations were available from 2022 to 2025, the model including interactions between calendar year and harmonic terms was considered exploratory.

Logistic regression was used to identify clinical factors associated with documented radiographic pneumonia. Covariates for the primary multivariable model were selected a priori based on clinical relevance and included age, male sex, continuous CRP concentration, abnormal auscultation, and chronic lung disease; variables were not selected solely on the basis of univariable statistical significance. A secondary model used CRP ≥ 2 mg/dL instead of continuous CRP. No imputation was performed, and multivariable analyses used complete cases. Model sample sizes and numbers of outcome events are reported. Temporal changes in documented radiographic pneumonia proportion were evaluated using crude and age- and sex-adjusted logistic models. Auscultation subtype models for severe pneumonia were adjusted for CRP ≥ 2 mg/dL and male sex. Receiver operating characteristic (ROC) analysis was used to characterize the discriminatory performance of CRP for documented radiographic pneumonia; the exploratory cutoff was selected using the Youden index. Results are expressed as odds ratios with 95% confidence intervals. Analyses were performed using Python and IBM SPSS Statistics version 25.

## 3. Results

### 3.1. Study Population and Care Setting

During the study period, 590 patients (including 192 adults and 398 children) tested positive for hMPV at Kyorin University Hospital. This study was designed to characterize adult hMPV infections, and the primary analyses were restricted to the 192 adult patients. Among them, 71 (37.0%) required hospitalization, and documented radiographic pneumonia was identified in 43 (60.6%) of those who were hospitalized. Pediatric hMPV-positive cases were not included in the main analysis; however, hospitalization was uncommon in this group, occurring in two of 398 patients (0.5%), with no pneumonia documented among the two hospitalized children.

**Figure 1:**
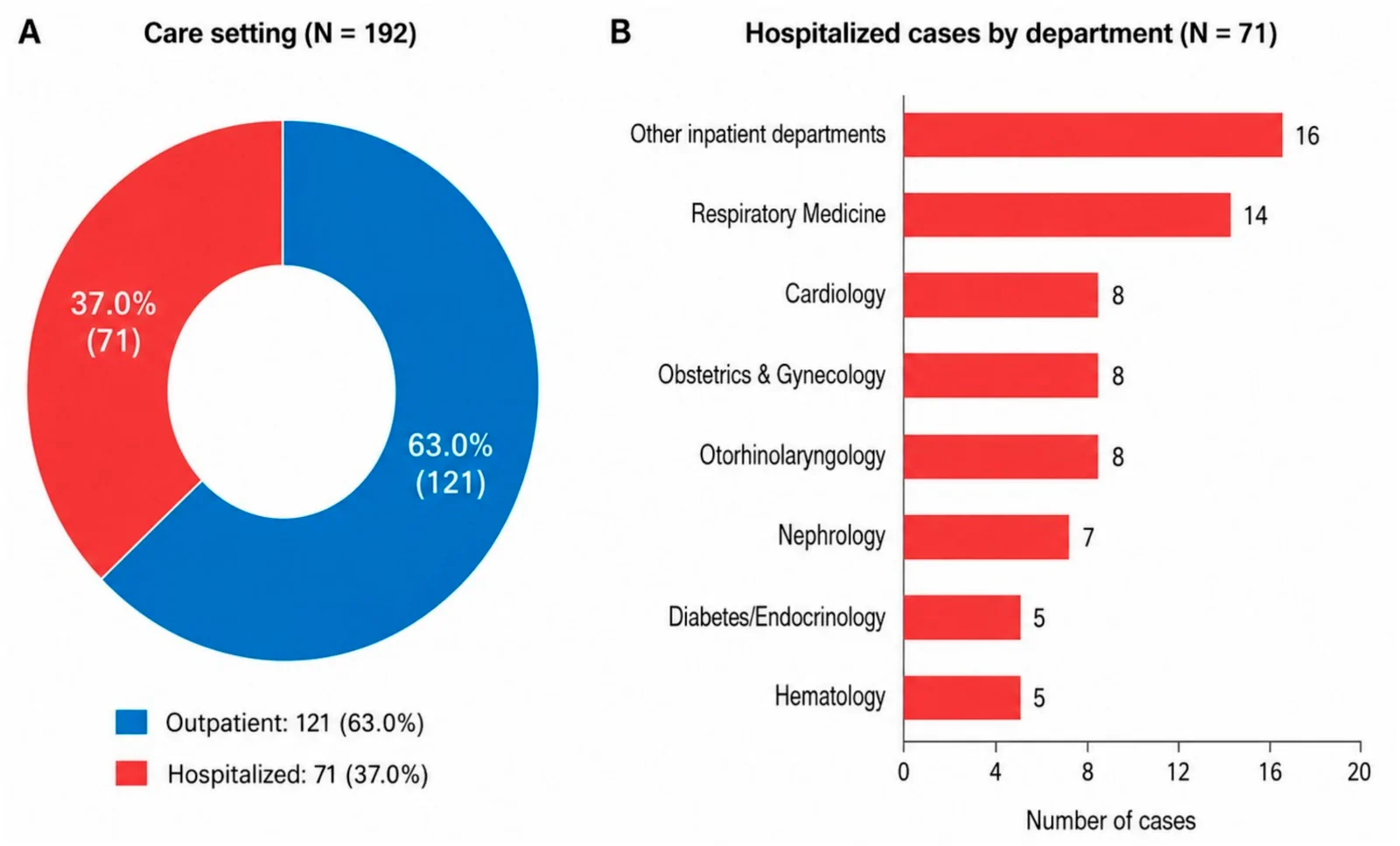
Distribution of adult patients with human metapneumovirus infection across care settings and admitting departments. (**A**) Care settings for adult patients with laboratory-confirmed human metapneumovirus (hMPV) infection diagnosed at a single center from December 2020 to February 2026. Of 192 adult hMPV-positive patients, 121 were managed as outpatients, and 71 were hospitalized. (**B**) Distribution of patients hospitalized for hMPV infection across admitting departments. Abbreviation: hMPV, human metapneumovirus.

Among the 192 adult patients, 121 were managed as outpatients, and 71 were hospitalized. Thus, 37.0% of adult patients with hMPV required inpatient care. Among hospitalized patients, the department with the most admissions was Respiratory Medicine, followed by Cardiology, Obstetrics and Gynecology, Otorhinolaryngology, Nephrology, Diabetes/Endocrinology, and Hematology. Other inpatient departments accounted for 16 hospitalized cases (Figure 1).

### 3.2. Annual and Seasonal Distribution

**Figure 2:**
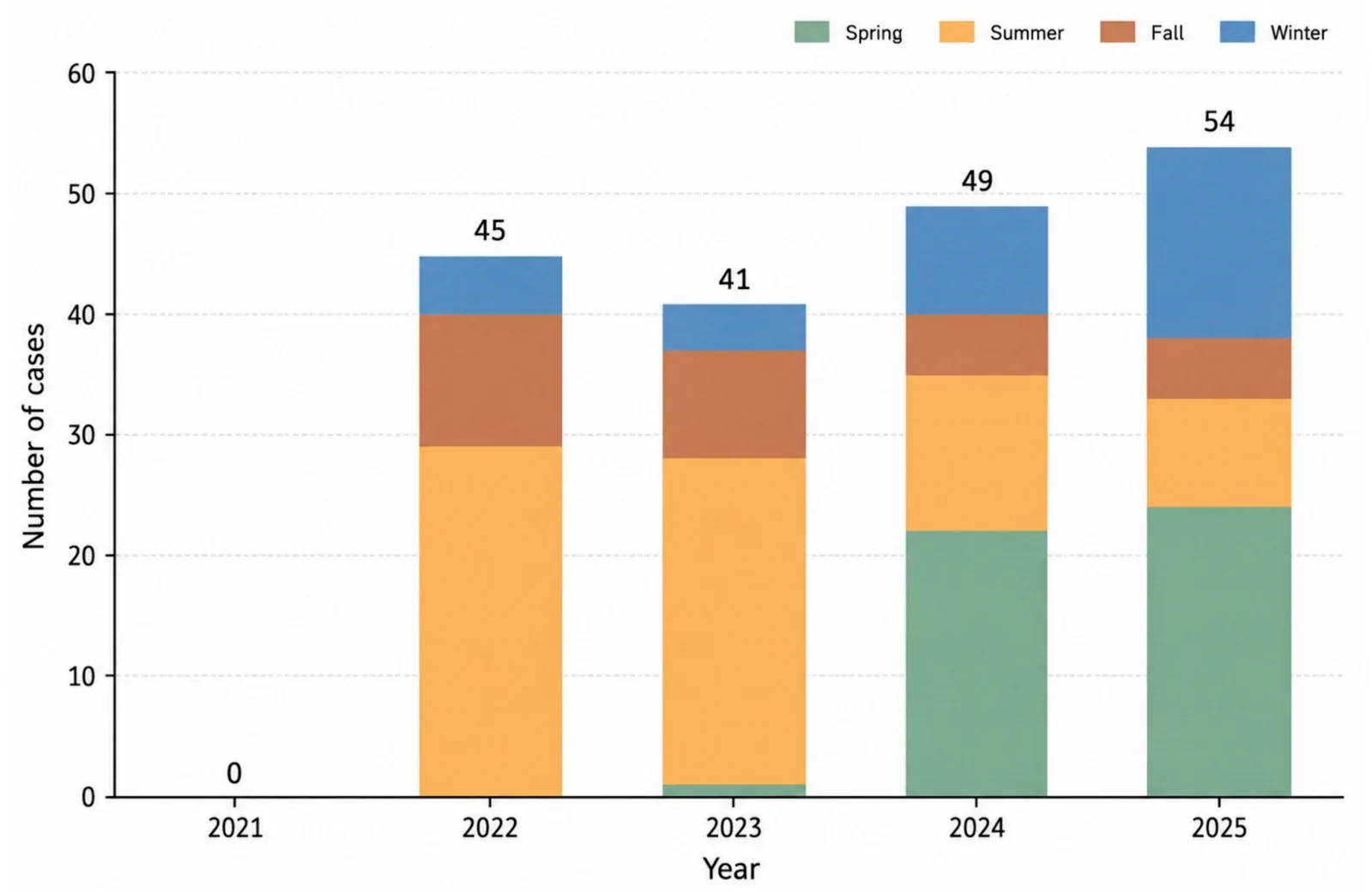
Annual and seasonal distribution of adult human metapneumovirus-positive cases from 2021 to 2025. Stacked bars show the number of adult patients with laboratory-confirmed human metapneumovirus (hMPV) infection across calendar years and seasons. Numbers above the bars indicate the annual total number of cases. No adult cases were detected in December 2020 or during 2021. Three adult cases detected in January 2026 were excluded from this figure and from the 2022–2025 temporal analyses because 2026 represented only a partial year. Abbreviation: hMPV, human metapneumovirus.

No adult hMPV-positive cases were detected in December 2020 or during 2021. Three adult cases were detected in January 2026. The annual numbers of adult hMPV-positive cases were 45, 41, 49, and 54 from 2022 to 2025, respectively, totaling 189 patients for the 2022–2025 temporal analyses. The seasonal distribution changed over time; cases were concentrated mainly in summer during 2022–2023, whereas spring and winter cases increased during 2024–2025 (Figure 2 and Supplementary Figure S1).

Season-specific Poisson regression of case counts showed a significant increase in spring and winter detections and a significant decrease in summer detections over time. The count ratio per year was 2.70 for spring (95% CI, 1.88–3.89; *p* < 0.001), 0.67 for summer (95% CI, 0.54–0.83; *p* < 0.001), 0.74 for fall (95% CI, 0.53–1.03; *p* = 0.076), and 1.61 for winter (95% CI, 1.16–2.24; *p* = 0.005) (Supplementary Table S1). Because no offset for testing volume was included, these estimates represent changes in observed institutional case counts rather than incidence rates.

Harmonic regression using seasonal counts from 2022 to 2025 demonstrated significant seasonality in adult hMPV detections (joint Wald test for sine and cosine terms; *p* < 0.001). An exploratory model including interactions between calendar year and harmonic terms suggested a time-varying seasonal pattern (likelihood ratio test, *p* < 0.001), consistent with a shift from summer predominance in 2022–2023 toward increased spring and winter detections in 2024–2025 (Supplementary Figure S2). Because this interaction model was based on only 16 season-year observations, the finding should be interpreted cautiously.

### 3.3. Temporal Changes in Documented Radiographic Pneumonia Burden

**Figure 3:**
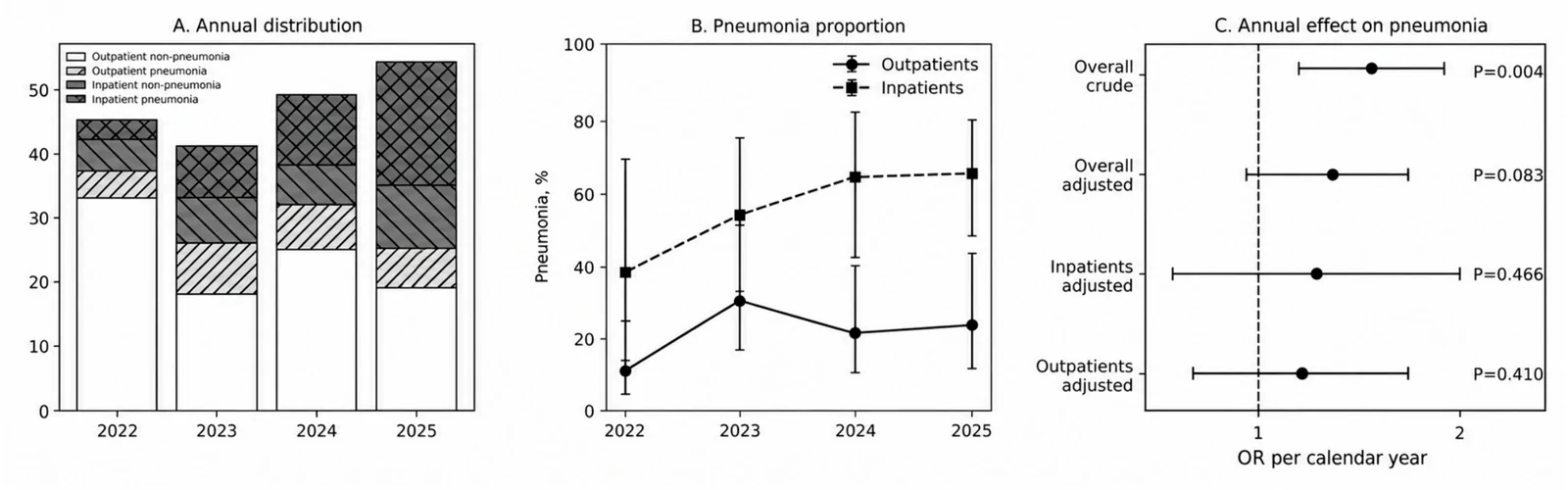
Temporal changes in institutional hMPV detection and documented radiographic pneumonia proportion among adults with human metapneumovirus infection. (**A**) Annual distribution of adult hMPV-positive cases across care settings and documented radiographic pneumonia statuses from 2022 to 2025. (**B**) Temporal trend in the proportion of documented radiographic pneumonia among outpatients and inpatients. Points indicate annual observed proportions, error bars indicate 95% confidence intervals, and shaded areas show fitted 95% confidence bands. (**C**) Crude and age- and sex-adjusted annual effects of calendar year on documented radiographic pneumonia proportion are shown separately for inpatients, outpatients, and the overall cohort. Estimates represent the change in odds of documented radiographic pneumonia per 1-year increase in calendar year. Error bars indicate 95% confidence intervals. The vertical reference line indicates an odds ratio of 1.0. Abbreviations: CI, confidence interval; hMPV, human metapneumovirus; OR, odds ratio. In this figure, pneumonia and non-pneumonia categories refer to documented radiographic pneumonia and no documented radiographic pneumonia, respectively.

The annual distribution of hMPV-positive cases stratified by care setting and documented radiographic pneumonia status showed that documented radiographic pneumonia was consistently more frequent among patients who were hospitalized than among outpatients. The proportion of outpatients with documented radiographic pneumonia was 10.8%, 30.8%, 21.9%, and 24.0% in 2022, 2023, 2024, and 2025, respectively. Among hospitalized patients, the proportion with documented radiographic pneumonia increased from 37.5% in 2022 to 65.5% in 2025 (Figure 3A,B).

Analyses of the annual effect of calendar year on documented radiographic pneumonia proportion showed a crude odds ratio of 1.51 per year (95% CI, 1.14–1.99; *p* = 0.004). After adjustment for age and sex, the overall odds ratio was 1.31 per year (95% CI, 0.97–1.77; *p* = 0.083). The adjusted odds ratio was 1.22 per year among inpatients (95% CI, 0.72–2.05; *p* = 0.466) and 1.19 per year among outpatients (95% CI, 0.79–1.78; *p* = 0.410) (Figure 3C).

### 3.4. Baseline Characteristics According to Documented Radiographic Pneumonia Status

Using the primary imaging definition, 68 adults were classified as having documented radiographic pneumonia and 124 as having no documented radiographic pneumonia. Chest radiography was performed in 110 patients (57.3%), chest CT in 81 (42.2%), and both in 76 (39.6%). Imaging modality use differed by documented radiographic pneumonia status (Supplementary Table S4); 77 patients with no documented radiographic pneumonia (62.1%) had no chest imaging, whereas 60 patients with documented radiographic pneumonia (88.2%) underwent both modalities. Compared with patients with no documented radiographic pneumonia, those with documented radiographic pneumonia were older, more often hospitalized, and more frequently had impaired activities of daily living, chronic lung disease, COPD, cardiovascular disease, diabetes mellitus, and chronic kidney disease. Dyspnea, sputum production, abnormal auscultation, hypoxemia, higher respiratory and heart rates, higher CRP, lactate dehydrogenase, aspartate aminotransferase (AST), blood urea nitrogen (BUN), and creatinine concentrations, and lower SpO_2_, hemoglobin, and albumin concentrations were also more common in patients with documented radiographic pneumonia (Table 1).

Among the patients who were hospitalized, the duration of stay was longer for those with documented radiographic pneumonia than for those without it (12 [9–16] vs. 6 [5–12] days, *p* = 0.007).

### 3.5. Clinical Factors Associated with Documented Radiographic Pneumonia

**Figure 4:**
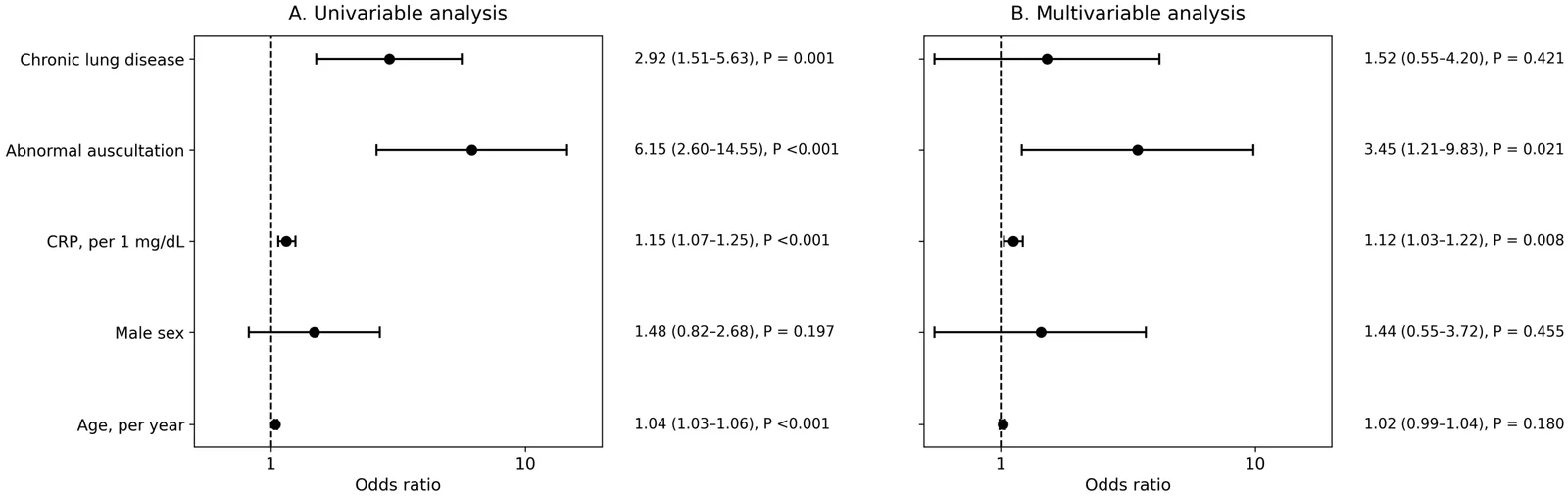
Clinical factors associated with documented radiographic pneumonia among adult human metapneumovirus-positive patients. (**A**) Univariable logistic regression analysis of clinical factors associated with documented radiographic pneumonia. (**B**) Primary multivariable logistic regression analysis including age, male sex, CRP concentration as a continuous variable, abnormal auscultation, and chronic lung disease. The complete-case cohort included 104 patients, of whom 62 had documented radiographic pneumonia. Error bars indicate 95% confidence intervals. The vertical dashed line indicates an odds ratio of 1.0. Abbreviations: CI, confidence interval; CRP, C-reactive protein; hMPV, human metapneumovirus; OR, odds ratio.

Univariable logistic regression showed associations of age, CRP concentration, abnormal auscultation, and chronic lung disease with documented radiographic pneumonia. The multivariable covariates were prespecified on clinical grounds rather than selected solely by univariable significance. CRP was unavailable in 70 patients and auscultation assessment was unavailable in 67; age, sex, and chronic lung disease status were available for all 192 patients. The complete-case cohort for the primary multivariable model included 104 patients, of whom 62 had documented radiographic pneumonia. In this model, higher CRP concentration (adjusted odds ratio per 1 mg/dL, 1.12; 95% CI, 1.03–1.22; *p* = 0.008) and abnormal auscultation (adjusted odds ratio, 3.45; 95% CI, 1.21–9.83; *p* = 0.021) remained independently associated with documented radiographic pneumonia (Figure 4). In the secondary model using CRP ≥ 2 mg/dL, CRP elevation (adjusted odds ratio, 8.36; 95% CI, 2.84–24.59; *p* < 0.001) and abnormal auscultation (adjusted odds ratio, 3.07; 95% CI, 1.04–9.08; *p* = 0.042) remained associated with documented radiographic pneumonia. Among 122 patients with CRP data, ROC analysis yielded an area under the curve of 0.786; the Youden index identified an optimal cutoff of 2.05 mg/dL (sensitivity, 87.9%; specificity, 60.7%). Accordingly, CRP ≥ 2 mg/dL was retained only as an exploratory threshold in secondary analyses (Supplementary Table S5). In the sensitivity analysis restricted to patients who underwent chest imaging, the complete-case cohort included 95 patients with 62 events; continuous CRP and abnormal auscultation remained associated with documented radiographic pneumonia (Supplementary Table S5).

### 3.6. Pathogen Profile and Documented Radiographic Pneumonia

Among the 192 adults with hMPV infection, 169 (88.0%) had hMPV alone, 11 (5.7%) had hMPV with other viruses, and 12 (6.3%) had hMPV with bacteria. Pathogen profiles differed according to documented radiographic pneumonia status (*p* = 0.001), and bacterial coinfection was more frequent in patients with documented radiographic pneumonia than in those with no documented radiographic pneumonia (10/68 [14.7%] vs. 2/124 [1.6%]) (Table 1). Clinical features according to pathogen profile are summarized in Table 2. Patients with bacterial coinfection were numerically more likely to require inpatient care (66.7%) and had a longer median hospital stay (16.5 days) than patients with hMPV alone or viral codetection, although these differences did not reach statistical significance (*p* = 0.076 and *p* = 0.081, respectively). Severe pneumonia occurred in 14/54 (25.9%) patients with hMPV alone, 0/2 (0.0%) with viral codetection, and 3/10 (30.0%) with bacterial coinfection (*p* = 0.674). Among the 12 patients with bacterial coinfection, three also had viral codetection and therefore represented multi-pathogen detections. Human rhinovirus was the most common co-detected virus (n = 6), followed by seasonal coronavirus, respiratory syncytial virus, and SARS-CoV-2 (n = 2 each). The detected bacterial pathogens are listed in Table 2.

### 3.7. Exploratory CRP and Auscultation-Based Classification

An exploratory 4-group classification based on CRP ≥ 2 mg/dL and abnormal auscultation showed differences in documented radiographic pneumonia frequency. The crude frequency was 20.7% with neither finding, 65.7% with CRP elevation only, 40.0% with abnormal auscultation only, and 88.6% with both findings. Using the “neither” group as the reference, age- and sex-adjusted odds ratios for documented radiographic pneumonia were 7.00 for CRP elevation alone (95% CI, 2.13–23.04; *p* = 0.001), 2.47 for abnormal auscultation alone (95% CI, 0.32–18.85; *p* = 0.383), and 26.50 for both findings (95% CI, 6.12–114.83; *p* < 0.001) (Supplementary Figure S3).

### 3.8. Auscultation Subtype and Severe Pneumonia

Among adult hMPV cases with documented radiographic pneumonia, crackle-predominant sounds were associated with severe pneumonia after adjustment for CRP ≥ 2 mg/dL and male sex (adjusted odds ratio, 6.00; 95% CI, 1.47–24.45; *p* = 0.012). Wheeze/rhonchi-predominant sounds were not significantly associated with severe pneumonia (adjusted odds ratio, 1.17; 95% CI, 0.17–7.98; *p* = 0.876) (Supplementary Table S2).

In the detailed category model, coarse crackles showed the strongest stable association with severe pneumonia (adjusted odds ratio, 8.84; 95% CI, 1.93–40.39; *p* = 0.005). Other estimates had wide confidence intervals or sparse events and should be interpreted as exploratory (Supplementary Table S3).

## 4. Discussion

hMPV infection is increasingly recognized as an important cause of adult acute respiratory infection [2,4,11]. In this single-center hospital-wide cohort spanning multiple departments, adult hMPV detections showed dynamic seasonal changes after the onset of the coronavirus disease 2019 (COVID-19) pandemic, shifting from summer predominance toward increased spring and winter detections. Radiographic pneumonia was common, especially among hospitalized patients, and abnormal auscultation and elevated CRP concentrations were independently associated with hMPV-associated documented radiographic pneumonia. These findings suggest that bedside assessment and routine laboratory testing can support decisions about chest imaging or closer follow-up in adults with hMPV infection.

The seasonal pattern observed in this study is consistent with reports describing hMPV activity in temperate climates from winter or early spring to late spring or early summer [12]. Recent surveillance has shown that the COVID-19 pandemic disrupted hMPV and RSV seasonality, with hMPV peak circulation returning in spring during recent seasons [13]. In our cohort, adult hMPV detections shifted from summer predominance in 2022–2023 toward increased spring and winter detections in 2024–2025. These findings suggest retained but time-varying seasonality, supporting continued surveillance as post-pandemic respiratory virus circulation evolves.

hMPV was initially recognized mainly as a pediatric pathogen, but older adults and immunocompromised patients are susceptible to pneumonia, hospitalization, asthma exacerbations, and COPD exacerbations [2,4,14,15,16,17,18,19,20]. In Japan, hMPV has been reported in adult asthma attacks, older inpatient and long-term care facility outbreaks, and adult community-acquired pneumonia [7,8,9,21,22,23]. By including both inpatients and outpatients with laboratory-confirmed hMPV infection, our study adds data from a broad adult clinical population rather than from a pneumonia-selected cohort. 

Abnormal auscultation was clinically informative. Prior adult studies reported wheezing, rales, and rhonchi in hMPV infection, with some suggesting more frequent wheezing than in influenza or RSV and others showing similar findings between hMPV and RSV [2,3,5,24]. In our study, abnormal auscultation was associated with documented radiographic pneumonia, and crackle-predominant sounds, particularly coarse crackles, were associated with severe pneumonia. This is clinically plausible because crackles may reflect parenchymal involvement more directly than wheezing or rhonchi. These exploratory findings support careful chest auscultation in adults with hMPV infection.

Elevated CRP was another robust marker of hMPV-associated documented radiographic pneumonia. This agrees with Japanese long-term care facility outbreak data showing higher white blood cell (WBC) count and CRP in hMPV pneumonia [7]. In our broader adult cohort, higher CRP concentration remained independently associated with documented radiographic pneumonia as a continuous variable. CRP ≥ 2 mg/dL was also associated with documented radiographic pneumonia in secondary analyses, but this dataset-derived threshold should be interpreted as exploratory until externally validated.

Co-detected pathogens also warrant consideration. Bacterial coinfection was uncommon but was enriched among patients with documented radiographic pneumonia. Patients with bacterial coinfection also showed numerically higher hospitalization frequency and longer hospital stays, although severe pneumonia did not differ significantly across pathogen profiles. In three patients, bacterial and viral codetections occurred together. Because hMPV, other viruses, and bacteria were detected concurrently in some patients, the leading pathogen could not be definitively determined; these findings should therefore be interpreted as associations with pathogen detection patterns rather than evidence of pathogen-specific causality. Larger studies are needed to clarify whether bacterial or multi-pathogen codetection modifies disease severity or clinical outcomes in adult hMPV infection.

This study has limitations. It was single-center and retrospective, and testing practices, imaging use, and admission thresholds may have influenced case detection and documented radiographic pneumonia classification. Because chest imaging was not uniformly obtained, especially in mildly ill outpatients, verification bias is possible and mild viral pneumonia with subtle CT findings may have been underdetected. Patients with no available chest radiography or CT result and no clinical diagnosis of pneumonia documented in the medical record were classified as having no documented radiographic pneumonia; some of these patients may therefore have had unrecognized mild pneumonia. We addressed this concern in a sensitivity analysis restricted to patients with available chest imaging, which yielded results consistent with the primary analysis. Testing volume was not standardized over the study period, and the Poisson models did not include an offset for the number of FilmArray tests or respiratory encounters; temporal estimates should therefore be interpreted as changes in institutional hMPV-positive case counts rather than population incidence. The exploratory harmonic interaction model was based on only 16 season-year observations and should be interpreted cautiously. The FilmArray respiratory panel does not detect all potentially relevant respiratory viruses, including human bocavirus, which can cause respiratory infection and pneumonia in adults [25]; viral codetection may therefore have been underestimated. Auscultation was extracted from routine records without a standardized protocol, so inter-physician variability cannot be excluded. Small event numbers limited subtype, severe pneumonia, and pathogen-profile analyses. External validation is needed before applying the CRP and auscultation-based approach broadly.

## 5. Conclusions

In conclusion, adult hMPV infection in this hospital-wide cohort was associated with substantial documented radiographic pneumonia burden and dynamic seasonal detection patterns after the onset of the COVID-19 pandemic. Elevated CRP concentrations and abnormal auscultation were independently associated with documented radiographic pneumonia, and crackle-predominant sounds were associated with severe pneumonia. These simple markers may help clinicians identify adults with hMPV infection who need careful evaluation for documented radiographic pneumonia and disease severity.

## Figures and Tables

**Table 1:** Baseline characteristics of adult hMPV-positive patients according to documented radiographic pneumonia status.

Characteristic	No Documented Radiographic Pneumonia (n = 124)	Documented Radiographic Pneumonia (n = 68)	*p*-Value
Demographics and care setting			
Age, years	49.0 [36.0–65.0]	74.0 [56.0–81.3]	<0.001
Male sex, n (%)	62 (50.0)	38 (55.9)	0.435
Inpatient care, n (%)	28 (22.6)	43 (63.2)	<0.001
ADL dependent, n (%)	18 (14.5)	24 (35.3)	<0.001
Comorbidities			
Lung disease, n (%)	29 (23.4)	28 (41.2)	0.010
COPD, n (%)	10 (8.1)	12 (17.6)	0.046
Asthma, n (%)	14 (11.3)	11 (16.2)	0.336
Cardiovascular disease, n (%)	20 (16.1)	22 (32.4)	0.009
Diabetes mellitus, n (%)	8 (6.5)	13 (19.1)	0.007
Chronic kidney disease, n (%)	5 (4.0)	8 (11.8)	0.041
Malignancy, n (%)	12 (9.7)	10 (14.7)	0.295
Clinical presentation			
Fever, n (%)	65 (52.4)	45 (66.2)	0.065
Dyspnea, n (%)	21 (16.9)	36 (52.9)	<0.001
Cough, n (%)	70 (56.5)	45 (66.2)	0.189
Sputum, n (%)	28 (22.6)	33 (48.5)	<0.001
Abnormal auscultation, n (%)	20 (16.1)	29 (42.6)	<0.001
Hypoxemia, n (%)	6 (4.8)	32 (47.1)	<0.001
Vital signs			
Body temperature, °C	37.2 [36.8–38.0]	37.8 [37.0–38.5]	0.06
Respiratory rate, /min	16.0 [16.0–18.0]	18.0 [17.0–20.0]	0.001
Heart rate, bpm	82 [72–95]	92 [80–105]	0.002
SpO_2_, %	97 [96–98]	93 [90–96]	<0.001
Laboratory findings			
WBC, /µL	7100 [5300–8950]	7150 [5800–9300]	0.337
Neutrophils, %	73.6 [61.4–80.5]	77.5 [65.5–82.4]	0.214
Lymphocytes, %	15.4 [10.0–21.9]	11.2 [8.7–18.0]	0.097
CRP, mg/dL	0.97 [0.46–4.41]	6.73 [2.90–11.91]	<0.001
LDH, U/L	217.0 [180.8–257.3]	243.5 [211.0–293.3]	0.004
Albumin, g/dL	4.0 [3.5–4.2]	3.7 [3.3–3.9]	0.007
AST, U/L	23.0 [18.0–31.0]	28.0 [22.3–37.0]	0.016
ALT, U/L	18.0 [12.0–27.8]	16.5 [13.0–27.8]	0.930
BUN, mg/dL	13.7 [10.1–17.8]	17.5 [11.7–26.9]	0.009
Creatinine, mg/dL	0.79 [0.65–0.94]	0.93 [0.70–1.29]	0.023
Hemoglobin, g/dL	13.5 [12.1–14.3]	12.5 [11.6–13.6]	0.013
Pathogen profile (FilmArray), n (%)			0.001
hMPV alone	113 (91.1)	56 (82.4)	
hMPV with other viruses	9 (7.3)	2 (2.9)	
hMPV with bacterial coinfection	2 (1.6)	10 (14.7)	

Notes: Data are shown as median [interquartile range] or n (%). *p*-values were calculated using the Mann–Whitney U test for continuous variables and the chi-square test or Fisher’s exact test for categorical variables, as appropriate. Documented radiographic pneumonia was defined as pneumonia-compatible pulmonary parenchymal abnormalities on chest radiography and/or chest CT. The no documented radiographic pneumonia group included 77 patients who did not undergo chest imaging and had no clinical diagnosis of pneumonia documented in the medical record; verification bias was addressed in a sensitivity analysis restricted to patients with available chest imaging. The *p*-value for pathogen profile represents the overall comparison across the three mutually exclusive categories. “hMPV with other viruses” indicates viral codetection without bacterial coinfection, whereas “hMPV with bacterial coinfection” indicates bacterial coinfection irrespective of viral codetection. ADL, activities of daily living; COPD, chronic obstructive pulmonary disease; CRP, C-reactive protein; CT, computed tomography; hMPV, human metapneumovirus; LDH, lactate dehydrogenase; SpO_2_, peripheral oxygen saturation; AST, aspartate aminotransferase; ALT, alanine aminotransferase; BUN, blood urea nitrogen; WBC, white blood cell count.

**Table 2:** Clinical features and co-detected pathogens according to pathogen profile among adult hMPV-positive patients.

Variable	hMPV Alone (n = 169)	hMPV with Other Viruses (n = 11)	hMPV with Bacteria (n = 12)	*p*-Value
Clinical characteristics				
Age, years	56.0 [39.0–75.0]	52.0 [38.0–64.5]	73.0 [69.0–80.2]	0.166
Male sex, n (%)	73 (43.2)	4 (36.4)	7 (58.3)	0.522
Inpatient care, n (%)	60 (35.5)	3 (27.3)	8 (66.7)	0.076
Documented radiographic pneumonia, n (%)	56 (33.1)	2 (18.2)	10 (83.3)	<0.001
Severe pneumonia, n/N (%)	14/54 (25.9)	0/2 (0.0)	3/10 (30.0)	0.674
Laboratory findings				
WBC, /µL	6900 [5600–8800]	9100 [6500–12,000]	8500 [6700–9900]	0.121
CRP, mg/dL	3.6 [0.8–8.0]	1.4 [0.8–7.4]	7.5 [5.1–11.0]	0.251
LDH, U/L	232.0 [197.2–277.2]	222.0 [187.0–304.0]	250.0 [222.0–310.5]	0.414
Albumin, g/dL	3.8 [3.4–4.1]	3.9 [3.5–4.1]	3.6 [3.1–3.8]	0.325
Outcome				
Length of stay among hospitalized patients, days	9.5 [6.0–15.0]	7.0 [4.5–16.0]	16.5 [11.8–21.0]	0.081

Notes: Data are shown as median [interquartile range] or n (%), unless otherwise indicated. Severe pneumonia was defined as an A-DROP score ≥3. *p*-values were calculated using the Kruskal–Wallis test for continuous variables and the chi-square test or Fisher’s exact test for categorical variables, as appropriate. Length of stay was evaluated among hospitalized patients. Pathogen categories were mutually exclusive. “hMPV with other viruses” indicates viral codetection without bacterial coinfection, whereas “hMPV with bacteria” indicates bacterial coinfection irrespective of viral codetection. Among the 12 patients with bacterial coinfection, three also had viral codetection and were included in the hMPV-with-bacteria group. Additional viruses detected included human rhinovirus (n = 6), seasonal coronavirus (n = 2), respiratory syncytial virus (n = 2), severe acute respiratory syndrome coronavirus 2 (SARS-CoV-2; n = 2), parainfluenza virus type 1 (n = 1), and parainfluenza virus type 3 (n = 1). The bacterial pathogens detected were *Haemophilus influenzae* (n = 2), *Mycoplasma pneumoniae* (n = 2), *Moraxella catarrhalis* (n = 2), *Klebsiella pneumoniae* (n = 2), *Pseudomonas* spp. (n = 1), *Escherichia coli* (n = 1), *Streptococcus pneumoniae* (n = 1), and methicillin-susceptible *Staphylococcus aureus* (n = 1). A-DROP, age, dehydration, respiratory failure, orientation disturbance, and blood pressure; CRP, C-reactive protein; hMPV, human metapneumovirus; LDH, lactate dehydrogenase; WBC, white blood cell count.

## Data Availability

The data presented in this study are not publicly available because they contain potentially identifiable patient information. Data may be available from the corresponding author on reasonable request and with permission from Kyorin University.

## Supplementary Materials

The following supporting information can be downloaded at: https://www.mdpi.com/article/10.3390/jcm15208025/s1.
