# Supplementary material for: Clinical Factors Associated with Documented Radiographic Pneumonia in Adults with Human Metapneumovirus Infection: A Hospital-Wide Cohort Study in Japan
Source: J Clin Med. 2026 Oct 16;15(20):8025. doi: 10.3390/jcm15208025 (PMC13706147; doi:10.3390/jcm15208025)
Supplement: Supplementary file 1 [file jcm-15-08025-s001.zip › jcm-4576952-supplementary.pdf]

Journal of
Clinical Medicine

Supplementary Figure Legends

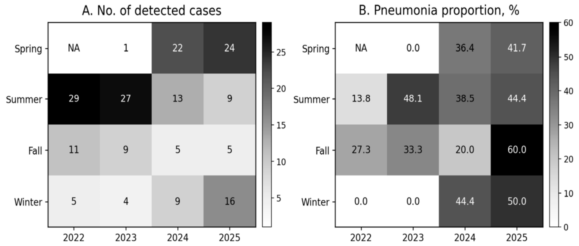

Supplementary Figure S1. Year–season heatmaps of adult human metapneumovirus-positive detections and proportion of documented radiographic pneumonia cases. (A) Heatmap showing the number of adult hMPV-positive cases across years and seasons from 2022 to 2025. (B) Heatmap showing the corresponding proportion of documented radiographic pneumonia cases across years and seasons. The value of the 2022 spring cell was set as "not applicable" because no cases were observed in that stratum. Data from 2026 were excluded because only data for January and February were available. Abbreviation: hMPV, human metapneumovirus.

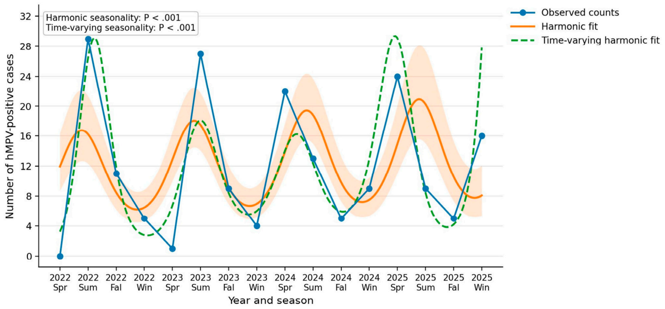

Supplementary Figure S2. Harmonic regression analysis of seasonal adult human metapneumovirus detections from 2022 to 2025. Observed seasonal counts of adult human metapneumovirus (hMPV)-positive cases are shown with harmonic regression fitted curves. The solid fitted curve represents the base harmonic Poisson model, including calendar year and annual sine/cosine terms. The dashed curve represents an exploratory time-varying harmonic model including interactions between calendar year and harmonic terms. Statistical significance for seasonality was assessed using a joint Wald test for sine and cosine terms, and time-varying seasonality was assessed using likelihood ratio testing of interaction terms. Because only 16 season-year observations were available, the interaction model should be interpreted as exploratory. Abbreviation: hMPV, human metapneumovirus.

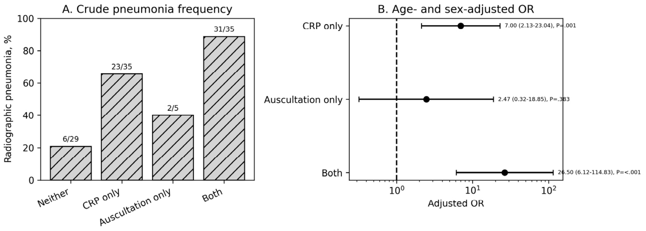

Supplementary Figure S3. Exploratory classification of documented radiographic pneumonia frequency according to CRP elevation and abnormal auscultation. (A) Crude documented radiographic pneumonia frequency among 4 groups defined by the presence or absence of CRP ≥2 mg/dL and abnormal auscultation. "Neither" indicates the absence of both CRP elevation and abnormal auscultation; "CRP only" indicates CRP ≥2 mg/dL without abnormal auscultation;

"auscultation only" indicates abnormal auscultation without CRP ≥2 mg/dL; and "both" indicates the presence of both findings. (B) Age- and sex-adjusted odds ratios for documented radiographic pneumonia using the "neither" group as the reference category. Error bars indicate 95% confidence intervals. Abbreviations: CI, confidence interval; CRP, C-reactive protein; OR, odds ratio.

Supplementary Table Legends

Supplementary Table S1. Season-specific temporal trends in adult hMPV-positive detections from 2022 to 2025.

Season	No. of detected cases, 2022–2025	Count ratio per year	95% CI	p-value
Spring	47	2.70	1.88–3.89	<.001
Summer	78	0.67	0.54–0.83	<.001
Fall	30	0.74	0.53–1.03	.076
Winter	34	1.61	1.16–2.24	.005

Notes: Count ratios were estimated using season-specific Poisson regression models of observed case counts, with calendar year modeled as a continuous variable. No offset for the number of FilmArray tests or respiratory encounters was included; therefore, estimates represent count ratios rather than incidence rate ratios. Statistical significance was assessed using the Wald test. The three adult cases detected in January 2026 were excluded because 2026 represented only a partial year. No adult hMPV-positive cases were observed in December 2020 or during 2021. CI, confidence interval; hMPV, human metapneumovirus.

Supplementary Table S2. Three-group auscultation model for severe pneumonia among adult hMPV-positive patients with documented radiographic pneumonia.

Auscultation category	Events/N	Adjusted OR (95% CI)	p-value
No abnormal auscultation	4/29	1.00 (Reference)	—
Crackle-predominant sounds	11/22	6.00 (1.47–24.45)	.012
Wheeze/rhonchi-predominant sounds	2/11	1.17 (0.17–7.98)	.876

Notes: The outcome was severe pneumonia, defined as an A-DROP score ≥3. Models were fitted among adult hMPV-positive patients with documented radiographic pneumonia and complete data for A-DROP score, auscultation category, CRP concentration, and sex (n = 62; severe pneumonia events = 17). Records without an auscultation assessment were treated as missing. Estimates were adjusted for CRP ≥2.0 mg/dL and male sex. The reference category was no abnormal auscultation. Crackle-predominant sounds included coarse crackles, fine crackles, coarse crackles/wheezes, and coarse crackles/rhonchi; any mixed finding containing crackles was assigned to the crackle-predominant category. Wheeze/rhonchi-predominant sounds included wheezes or rhonchi without crackles. A-DROP, age, dehydration, respiratory failure, orientation disturbance, and blood pressure; CI, confidence interval; CRP, C-reactive protein; hMPV, human metapneumovirus; OR, odds ratio.

Supplementary Table S3. Detailed auscultation category model for severe pneumonia among adult hMPV-positive patients with documented radiographic pneumonia.

Auscultation category	Events/N	Adjusted OR (95% CI)	p-value
No abnormal auscultation	4/29	1.00 (Reference)	—
Coarse crackles	9/15	8.84 (1.93–40.39)	.005
Fine crackles	0/3	Not estimable	—
Wheezes	1/6	1.13 (0.10–13.14)	.922
Rhonchi	1/5	1.20 (0.10–14.55)	.885
Coarse crackles/wheezes	1/3	3.15 (0.21–47.13)	.407
Coarse crackles/rhonchi	1/1	Not estimable	—

Notes: The outcome was severe pneumonia, defined as an A-DROP score ≥3. Models were fitted among adult hMPV-positive patients with documented radiographic pneumonia and complete data for A-DROP score, auscultation category, CRP, and sex (n = 62; severe pneumonia events = 17). Records without an auscultation assessment were treated as missing. Estimates were adjusted for CRP ≥2.0 mg/dL and male sex. The reference category was no abnormal auscultation. Estimates for sparse categories should be interpreted cautiously; fine crackles and coarse crackles/rhonchi were not estimated because of zero-event or single-case cells. A-DROP, age, dehydration, respiratory failure, orientation disturbance, and blood pressure; CI, confidence interval; CRP, C-reactive protein; hMPV, human metapneumovirus; OR, odds ratio.

Supplementary Table S4. Imaging assessment according to documented radiographic pneumonia status among adult hMPV-positive patients.

Imaging assessment	No documented radiographic pneumonia (n = 124)	Documented radiographic pneumonia (n = 68)	p-value
Chest radiography performed	45 (36.3)	65 (95.6)	<.001
Chest CT performed	18 (14.5)	63 (92.6)	<.001
Both chest radiography and CT	16 (12.9)	60 (88.2)	<.001
Chest radiography only	29 (23.4)	5 (7.4)	.005
Chest CT only	2 (1.6)	3 (4.4)	.348
No chest imaging performed	77 (62.1)	0 (0.0)	<.001
Pneumonia-compatible CXR findings	0 (0.0)	56 (82.4)	<.001
Pneumonia-compatible CT findings	0 (0.0)	61 (89.7)	<.001
Any abnormal imaging finding	3 (2.4)	68 (100.0)	<.001

Notes: Data are shown as n (%). Chest radiography and CT findings were assessed separately. For each modality, the examination was considered not to have been performed when no imaging result was available in the chart abstraction. When the examination had been performed but no abnormal findings were documented, it was classified as imaging performed without abnormal findings. Documented radiographic pneumonia was defined by pneumonia-compatible pulmonary parenchymal abnormalities on chest radiography and/or chest CT. Patients with no available chest radiography or CT result and no clinical diagnosis of pneumonia documented in the medical record were included in the no documented radiographic pneumonia group. Pneumonia-compatible findings included infiltrates, nodular or ground-glass opacities, consolidation, tree-in-bud nodules, or combinations of these findings. Isolated pleural effusion, isolated bronchial wall thickening, and isolated linear opacities were not classified as pneumonia-compatible findings in the primary definition. p-values were calculated using Fisher's exact test. CT, computed tomography; CXR, chest radiography; hMPV, human metapneumovirus.

Supplementary Table S5. Secondary and sensitivity analyses of clinical factors associated with documented radiographic pneumonia among adult hMPV-positive patients.

Model / variable	Complete-case cohort	Adjusted OR (95% CI)	p-value
Primary analysis; CRP as continuous variable	**104 (62 events)**		
CRP, per 1 mg/dL		1.12 (1.03–1.22)	.008
Abnormal auscultation		3.45 (1.21–9.83)	.021
**Secondary model; CRP ≥2.0 mg/dL**	**104 (62 events)**		
CRP ≥2.0 mg/dL		8.36 (2.84–24.59)	<.001

Abnormal auscultation		3.07 (1.04–9.08)	.042
**Sensitivity: any abnormal CXR/CT finding; CRP as continuous variable**	**104 (65 events)**		
CRP, per 1 mg/dL		1.11 (1.02–1.21)	.013
Abnormal auscultation		3.43 (1.15–10.22)	.027
Sensitivity: patients with available chest imaging only; CRP as continuous variable	**95 (62 events)**		
CRP, per 1 mg/dL		1.16 (1.03–1.30)	.011
Abnormal auscultation		4.22 (1.30–13.70)	.017

Notes: All models included age, male sex, chronic lung disease, abnormal auscultation, and either continuous CRP concentration or CRP ≥2.0 mg/dL, as indicated. Only estimates for CRP and abnormal auscultation are shown. No imputation was performed; all models used complete cases. The primary definition required pneumonia-compatible pulmonary parenchymal abnormalities on chest radiography and/or chest CT. A sensitivity analysis restricted to patients with available chest imaging was performed to address verification bias. In a separate sensitivity analysis using any abnormal chest imaging finding as an alternative definition, two additional patients with isolated bronchial wall thickening on CT were classified as having documented radiographic pneumonia. CI, confidence interval; CRP, C-reactive protein; CT, computed tomography; CXR, chest radiography; hMPV, human metapneumovirus; OR, odds ratio.
